# MiRNA Polymorphisms and Hepatocellular Carcinoma Susceptibility: A Systematic Review and Network Meta-Analysis

**DOI:** 10.3389/fonc.2020.562019

**Published:** 2021-01-19

**Authors:** Qimeng Zhang, Xueying Xu, Mingcheng Wu, Tiantian Qin, Shaoning Wu, Hongbo Liu

**Affiliations:** Department of Health Statistics, School of Public Health, China Medical University, Shenyang, China

**Keywords:** hepatocellular carcinoma, microRNA, polymorphism, network meta-analysis, susceptibility, hepatitis B virus

## Abstract

**Background:**

Hepatocellular carcinoma (HCC) is an intractable public health threat worldwide, representing the second leading cause of cancer-related mortality, with limited early detection and therapeutic options. Recent findings have revealed that the susceptibility of HCC is closely related to microRNA (miRNA). We performed this systematic review with a network meta-analysis to investigated four single nucleotide polymorphisms (SNPs) that most regularly reported in miRNAs, exploring their involvement in HCC susceptibility and interaction with hepatitis B virus (HBV).

**Methods:**

Databases were reviewed for related studies published up to May 2019 to identify all studies that compared genotypes of miR-146a rs2910164, miR-149 rs2292832, miR-196a2 rs11614913, and miR-499 rs3746444 with no language and date restrictions. A pairwise meta-analysis was performed to estimate pooled odds ratios and 95% confidence intervals incorporating heterogeneity to assess the relationship between four miRNA polymorphisms and HCC. To further clarify the effect of polymorphisms on HCC, a Bayesian network meta-analysis was conducted to combine the effective sizes of direct and indirect comparisons. Calculations were performed by R version 3.6.1 and STATA 14.0. All steps were performed according to PRISMA guidelines.

**Results:**

A total of 20 studies were enrolled in this network meta-analysis, providing 5,337 hepatocellular carcinoma cases and 6,585 controls. All included studies had an acceptable quality. Pairwise meta-analysis demonstrated that miR-196a2 rs11614913 was significantly associated with the susceptibility of HCC, while the other three SNPs were not found to have a significant association. In the analysis of HCC patients under different HBV infection status, only miR-196a2 revealed correlation of threefold risk. The network results showed no significant difference in the distribution of genotype frequencies except for miR-196a2, which appeared to have the highest superiority index when comparing and ranking four SNPs.

**Conclusion:**

MiR-196a2 rs11614913 was significantly associated with the susceptibility of HCC, especially for HBV- related HCC, and that individuals with TC/CC were more susceptible. No significant association was found in the other three miRNA genes. MiR-196a2 could serve as the best predictor of susceptibility in HCC.

## Introduction

Hepatocellular carcinoma (HCC), the most common form of primary liver cancer, has been an intractable public health threat worldwide (1). Approximately 700,000 new cases and 600,000 deaths are attributable to HCC annually (2), representing the sixth leading cause of cancer and the second leading cause of cancer-related mortality (3). Asia, Sub-Saharan Africa and the Middle East are high-risk regions with high incidence rates of HCC (4), and in some of these regions HCC ranks as the leading cause of death due to cancer (5). But, it is worth noting that the incidence and mortality have been increasing in North America and some areas of Europe (6, 7). The incidence in the United States has tripled over the past three decades (8). In the European Union, estimated by WHO, about 47,000 people die of liver cancer each year (9). In Canada, HCC has become the only cancer whose mortality rate is still on the rise. The incidence has been increasing rapidly and is projected to continue beyond 2020 (3). Multiple factors are responsible for its development including inborn diseases, chemicals, and viruses, of which Hepatitis B virus (HBV) is widely acknowledged. HCC has become a tremendous global burden (10), with the characteristics of high incidence, short duration, poor prognosis, high degree of malignancy, and five-year survival rate of 7% (11), yet remains one of the most ill-informed cancers and compounds by limited early detection and therapeutic options (12, 13). Therefore, exploring and clarifying the disease mechanism of HCC is conducive for effective prevention and treatment (14).

Genetic association studies are of great significance for epidemiological analyses, as they can identify candidate genome regions associated to specific diseases (15). Many findings have revealed that the presence of single nucleotide polymorphisms (SNPs) in some miRNA genes can alter the expression or maturation of miRNAs, making individuals more susceptible to certain types of cancer (16, 17). Several SNPs in miRNA genes can influence the development of HCC (18), providing a novel perspective of pathophysiological mechanism for the etiology of HCC (19).

MiR-146a (rs2910164), miR149 (rs2292832), miR-196a2 (rs11614913), and miR-499 (rs3746444) are well-established functional miRNAs (20–24). Researches have demonstrated that they can participate in essential regulatory processes related to cellular senescence, inflammation, immune response thus have potential value as biomarkers for many diseases (25, 26). The results on the association between genetic polymorphisms and HCC susceptibility remain inconsistent due to differences in race, disease stage, sample size, or other uncertainties. To further explore whether polymorphisms in these four SNPs might predispose to HCC, additional research and quantitative statistical studies are required to resolve discrepancies (27). Network meta-analyses (NMA) can be used to summarize and compare studies on multiple interventions (28), and combine direct and indirect evidence thus produce a result more precise (29). We conducted this systematic review with a network meta-analysis to provide more comprehensive information on the polymorphisms of four selected miRNAs and their involvement in HCC susceptibility and interaction with HBV.

## Materials and Methods

### Literature Search

In this systematic review and meta-analysis, we searched the database of PubMed, EMbase and the Cochrane Central Register to identify all eligible case-controlled trials that compared genotypes of the four selected miRNA genes in HCC patients with non-cancer control groups. All searches were performed in May 2019 and no language and date restrictions were set. The searching items were: “hepatocellular carcinoma”, “hepatoma”, “liver cancer”, “HCC”, and “microRNAs”, “miRNA”, and “polymorphism”, “allele”, “variation”, “SNP”.

### Selection Criteria

Eligible studies met the following criteria: (1) Case-controlled trials of subjects with HCC and healthy participants without HCC; (2) Evaluate the relationship between four common SNPs of miRNA (miR-146a rs2910164, miR-149 rs2292832, miR-196a2 rs11614913, miR-499 rs3746444) and HCC risk; (3) Investigate at least two selected SNPs at the same time; (4) Either DNA sequencing or PCR is used as a genotypic method for detection.

Articles were excluded based on the criteria: (1) Duplicated articles or data; (2) Irrelevant cancers or SNPs; (3) Functional studies; (4) Lack of available genotype frequency.

### Data Abstraction and Assessment of Bias

Two investigators independently abstracted the data on the studies. Discrepancies were resolved by consensus, referring back to the original study, or consulting a third reviewer. Besides genotype and frequency, the following data were also extracted from original studies: first author, year of publication, country, ethnicity, genotyping method, study design, case-control matching, sample size (cases/controls), and HBV infection status. To reduce the risk of bias due to individual studies, the Newcastle-Ottawa scale (NOS) score was applied to evaluate the methodological quality. The scale assesses three domains (selection bias, group comparability, and cohort exposure), based on “yes” or “no” answers to the following questions: (1) Is the case definition adequate; (2) Is there representativeness of the cases; (3) Is there selection of controls; (4) Is there a definition of controls; (5) Is there comparability of cases and controls; (6) Is there ascertainment of exposure; (7) Is the same method of ascertainment used for cases and controls; (8) Is there a non-response rate. The total score of NOS ranges from 0 to 9. A systematic analysis of the included studies was performed, and those with scores less than 5 were excluded. Two investigators independently performed the risk of bias assessments, with disagreement resolved by a third researcher when needed.

### Statistical Analysis

A traditional pairwise meta-analysis was performed to estimate pooled odds ratios (ORs) and 95% confidence intervals (CIs) incorporating heterogeneity within and between studies. Statistical heterogeneity between each study was assessed with using the Chi-square tests and the inconsistency index I-square, with the values of 25%, 50%, and 75% denoting low, moderate and high heterogeneity, respectively. A random effects model was applied when *I^2^* are over 50% (30). We went a step further and analyzed whether different genotypes might predispose to HCC under different HBV infection status. Meta-regression analysis was performed on the basis of the ethnicity, HWE, case-control match, and sample size to assess the heterogeneity that may have influences on the association between miRNA polymorphisms and HCC. The Begg’ s and Egger’ s tests were conducted to detect potential publication bias. Calculations and plotting were implemented by STATA 14.0 software.

To further clarify the effect between four polymorphisms of miRNA on HCC, we conducted a Bayesian network meta-analysis. First a network plot depicting the connection within four SNPs was drawn. Every SNP was represented by a node, and the node size represented the number of studies of a corresponding SNP, the line thickness between two nodes represented the number of paired studies. Then, the analysis of variance (ANOVA) model was applied to combine the effective sizes of direct and indirect comparisons. The ability to rank interventions is an attractive feature of NMA compared to traditional analysis. The superiority index was calculated to rank competing polymorphisms. The superiority index ranges from 0 to ∞, which tends toward ∞ as the genetic model has a higher likelihood of predicting the risk of HCC and tends toward 1 indicating equal effect. Calculations were performed by R version 3.6.1 and STATA 14.0 was used to assist graphical functions.

## Results

### Characteristics and Bias of Enrolled Studies

**Figure 1:**
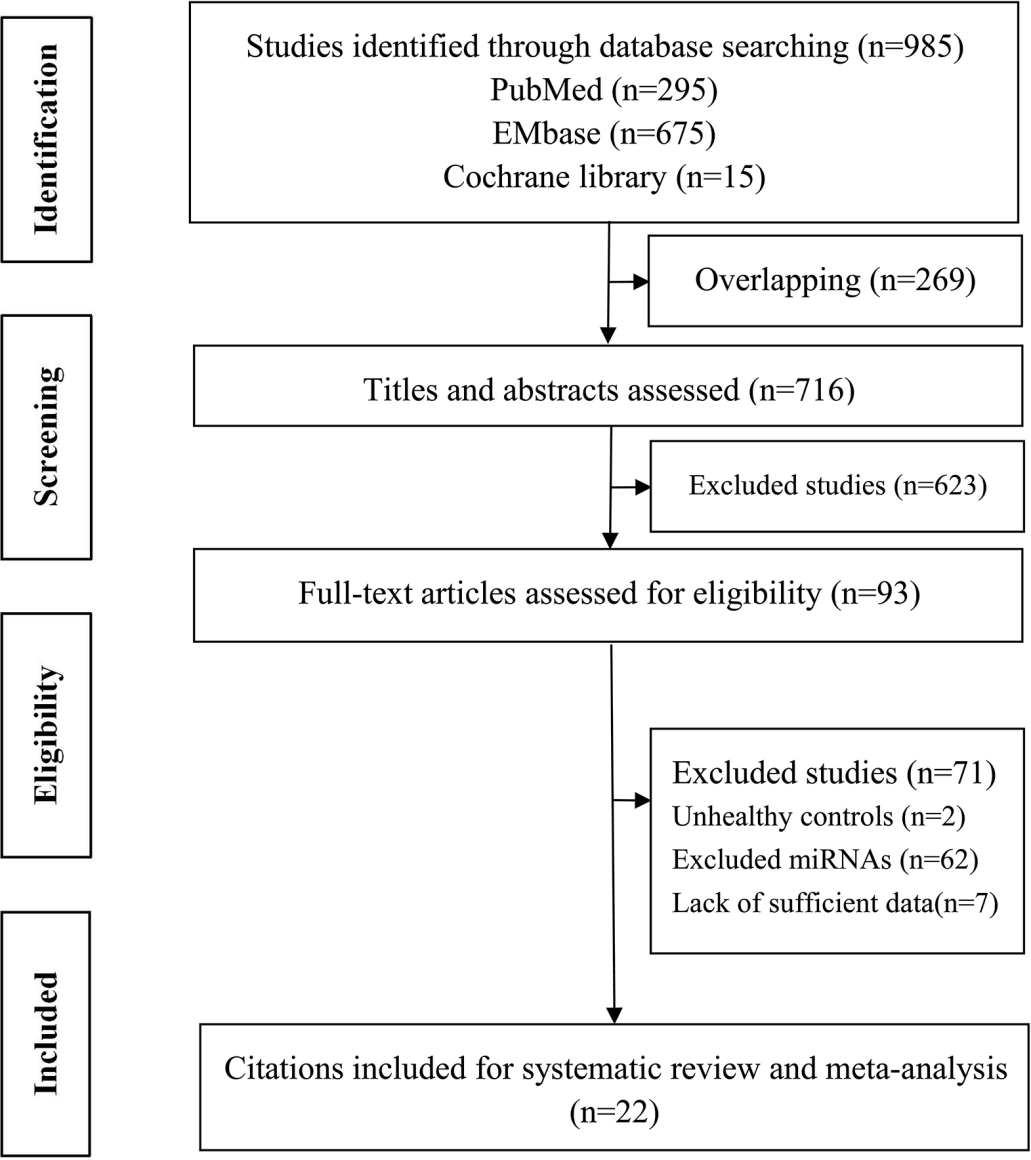
PRISMA Flow diagram of the literature during the review process for the systematic review and meta-analysis.

**Figure 2:**
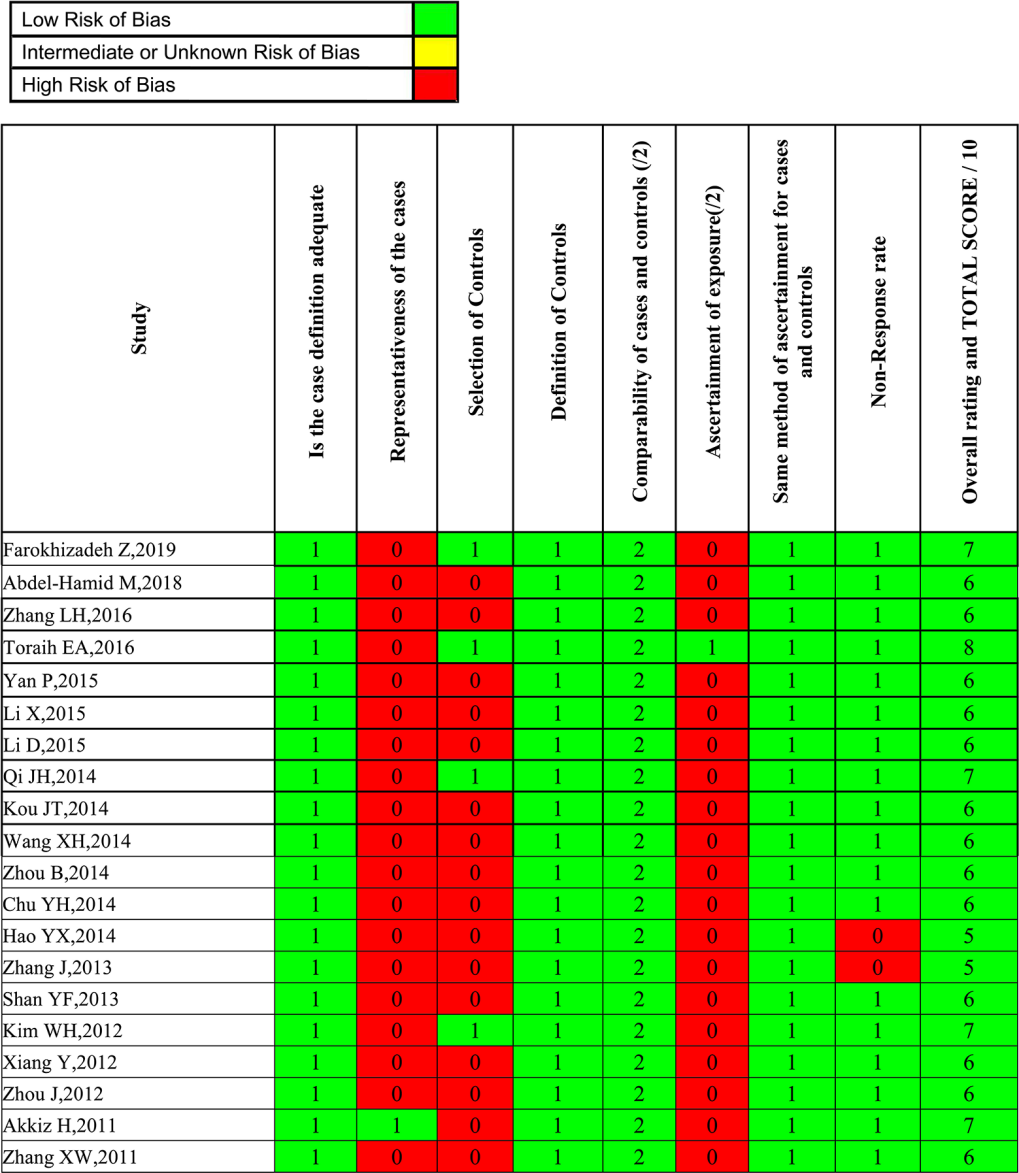
Risk of bias assessment using the Newcastle-Ottawa Scale for case-control studies.

Overall, 985 citations were identified using the search strategy. Among them, 269 citations were duplicates and 623 were excluded due to inappropriate tumor, functional studies, meta-analysis, reviews after assessing titles and abstracts. In the remaining 93 articles, there are two unhealthy controls, seven lack of sufficient data, 62 articles of irrelevant polymorphisms were removed. In Akkiz’s study, three articles investigating the same population and separately reporting three miRNA genes were considered as one. Therefore, 20 studies were enrolled providing a total of 5,337 HCC cases and 6,585 controls (**Figure 1**). The publication date of enrolled studies was from 2011 to 2019. The publications were mostly conducted in Asia, and two from Africa. In terms of ethnicity, 16 of the studies had Asian subjects and four studies had Caucasian subjects. Characteristics of included studies were presented in **Table 1**. The assessments of study quality were presented in **Figure 2**, and the NOS scale score result showed that all included studies had an acceptable quality.

**Table 1:** Main characteristics and methodological quality of eligible studies.

Author	Year	Country	Continent	Ethnicity	Genotyping method	Source of controls	Match	Case/Control
Farokhizadeh et al. (31)	2019	Iran	Asia	Caucasian	PCR-RFLP	PB	Y	100/120
Abdel-Hamid et al. (32)	2018	Egypt	Africa	Caucasian	PCR-RFLP	PB	Y	50/50
Zhang et al. (33)	2016	China	Asia	Asian	PCR-RFLP	HB	N	175/302
Toraih et al. (34)	2016	Egypt	Africa	Caucasian	Real-time PCR	PB	Y	60/150
Yan et al. (35)	2015	China	Asia	Asian	PCR-RFLP	HB	N	274/328
Li et al. (36)	2015	China	Asia	Asian	PCR-RFLP	HB	N	266/266
Li et al. (37)	2015	China	Asia	Asian	PCR-RFLP	HB	Y	184/184
Qi et al. (38)	2014	China	Asia	Asian	Sequenom	PB	N	314/407
Kou et al. (39)	2014	China	Asia	Asian	PCR-RFLP	HB	N	271/532
Wang et al. (40)	2014	China	Asia	Asian	PCR-RFLP	HB	N	152/304
Zhou et al. (41)	2014	China	Asia	Asian	Sequenom	HB	N	266/281
Chu et al. (42)	2014	China	Asia	Asian	PCR-RFLP	HB	Y	188/337
Hao et al. (43)	2014	China	Asia	Asian	PCR-RFLP	HB	Y	235/281
Zhang et al. (44)	2013	China	Asia	Asian	Sequenom	HB	N	1,000/1,000
Shan et al. (45)	2013	China	Asia	Asian	PCR-RFLP	HB	Y	172/185
Kim et al. (46)	2012	Korea	Asia	Asian	PCR-RFLP	PB	N	159/201
Xiang et al. (47)	2012	China	Asia	Asian	PCR-RFLP	HB	N	100/100
Zhou et al. (48)	2012	China	Asia	Asian	PCR-RFLP	HB	Y	186/483
Akkiz et al. (49–51)	2011	Turkey	Asia	Caucasian	PCR-RFLP	HB	Y	222/222
Zhang et al. (52)	2011	China	Asia	Asian	PIRA-PCR	HB	N	963/852

PCR, polymerase chain reaction; RFLP, restriction fragment length polymorphism assay; PB, population-based; HB: hospital-based.

### Pairwise Meta-Analysis

**Figure 3:**
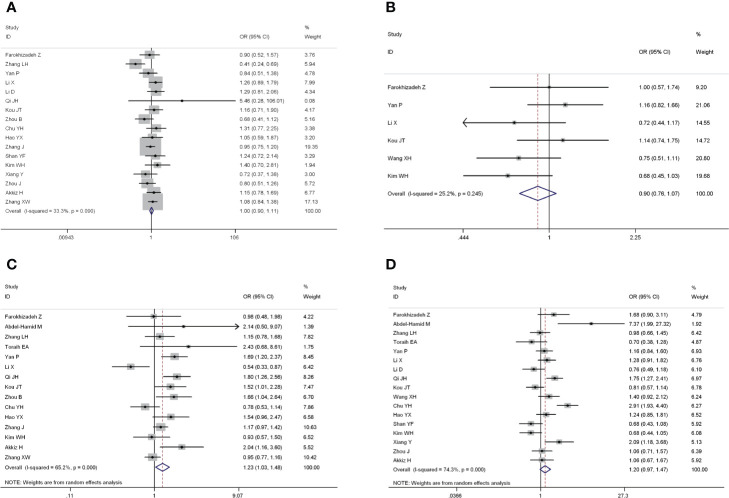
Forest plots of the association of four single nucleotide polymorphisms (SNPs) and hepatocellular carcinoma (HCC) risk. **(A)** miR-146a rs2910164; **(B)** miR-149 rs2292832; **(C)** miR-196a2 rs11614913; **(D)** miR-499 rs3746444.

**Figure 4:**
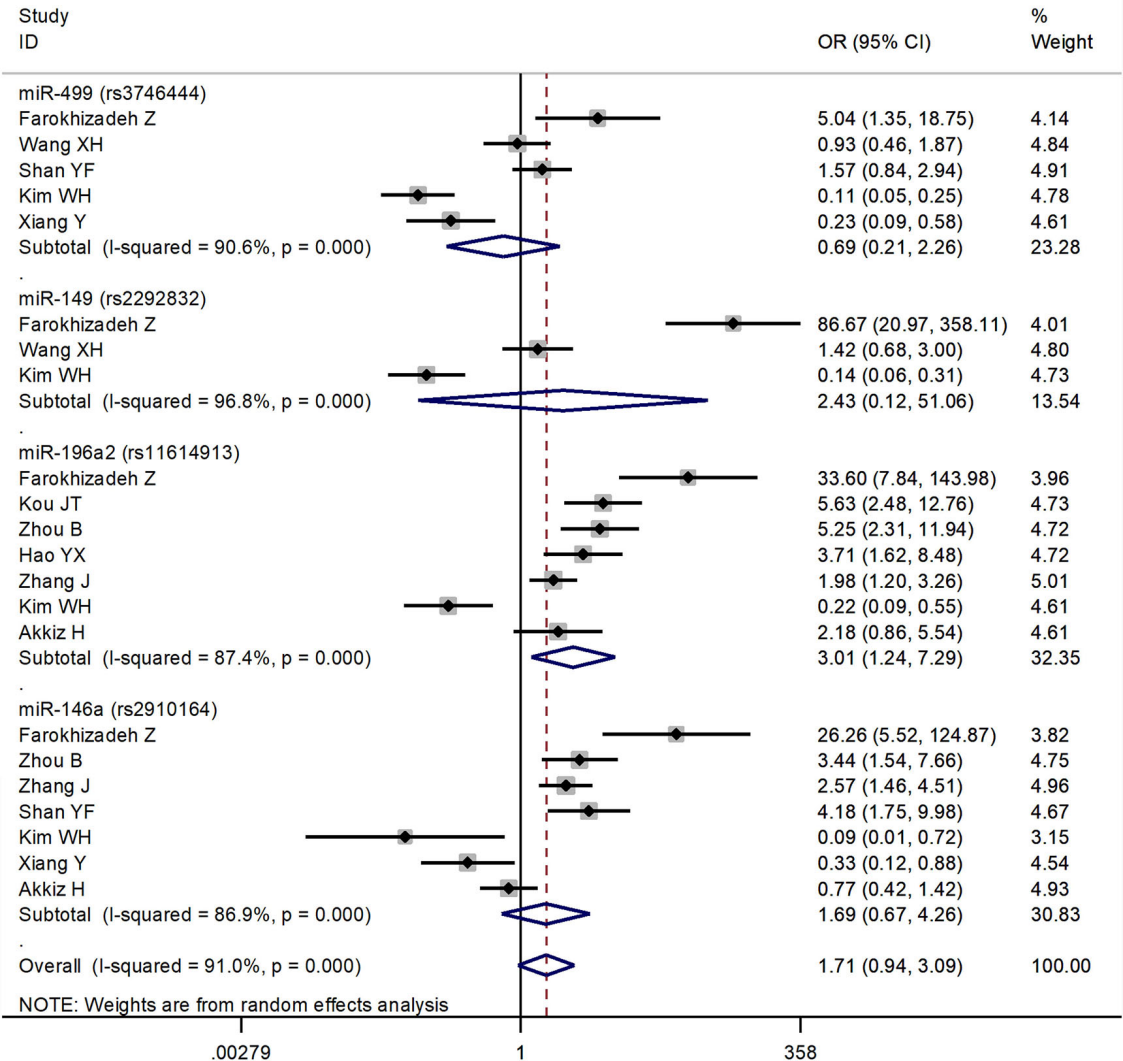
Forest plots of the association of four single nucleotide polymorphisms (SNPs) under different hepatitis B virus (HBV) infection status in hepatocellular carcinoma (HCC) patients.

**Figure 5:**
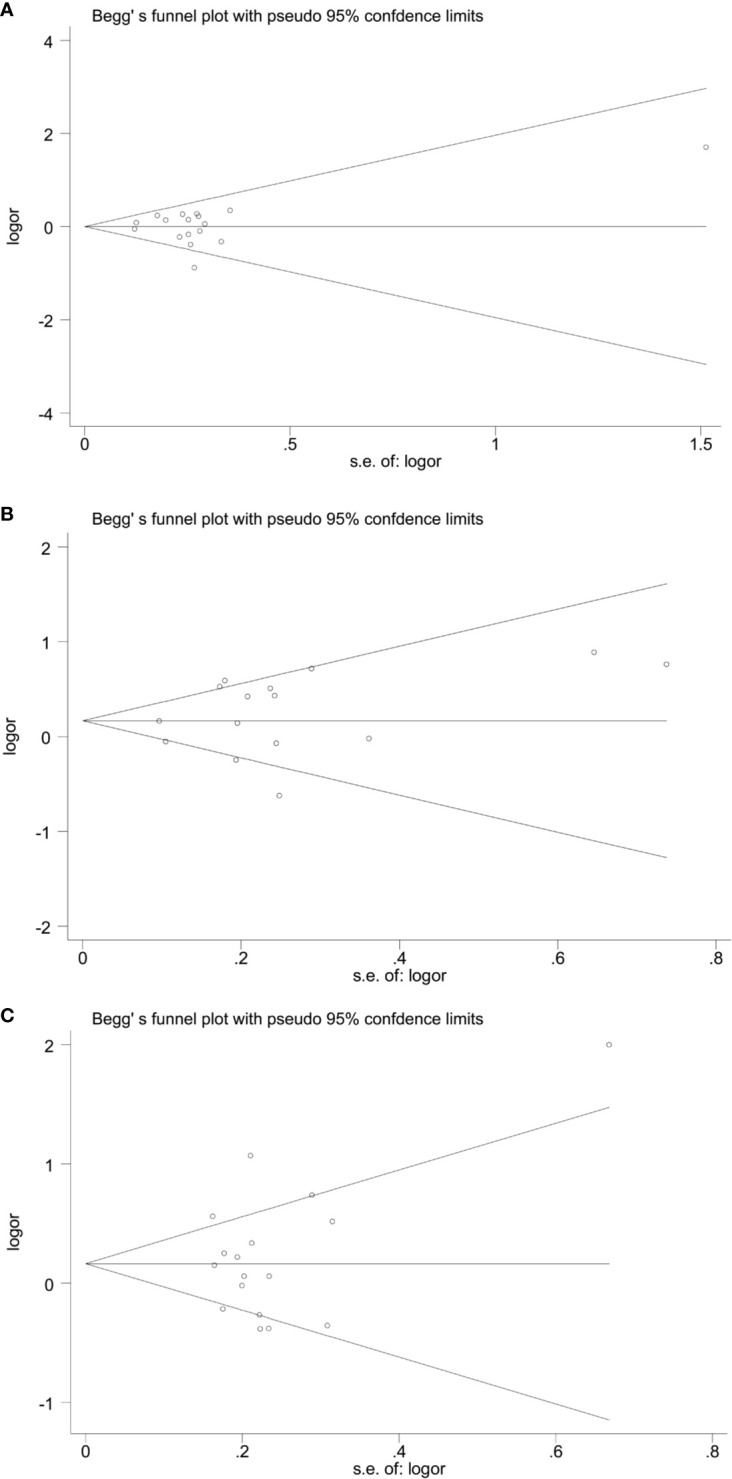
Begg’ funnel plots of publication bias. **(A)** miR-146a rs2910164; **(B)** miR-196a2 rs11614913; **(C)** miR-499 rs3746444.

The forest plots of four miRNAs and their involvement in HCC susceptibility and relationship with HBV were explored and compared in **Figures 3** and **4**, respectively. The results indicated that miR-196a2 rs11614913 polymorphism was significantly associated with the susceptibility of HCC (miR-196a2 rs11614913: TC+CC vs. TT: OR=1.232, 95%CI=1.028–1.476), while the other three SNPs were not found to have a significant association (miR-146a rs2910164: GC+CC vs. GG: OR=1.003, 95%CI=0.904–1.113; miR-149 rs2292832: TC+CC vs. TT: OR=0.898, 95%CI=0.756–1.068; miR-499 rs3746444: TC+CC vs. TT: OR=1.197, 95%CI= 0.973–1.472, respectively). In the analysis of HCC patients under different HBV infection status, only miR-196a2 rs11614913 revealed significant correlation of threefold risk (miR-146a rs2910164: GC+CC vs. GG: OR=1.687, 95%CI=0.667–4.263; miR-149 rs2292832: TC+CC vs. TT: OR=2.435, 95%CI=0.116–51.063; miR-196a2 rs11614913: TC+CC vs. TT: OR=3.005, 95%CI=1.239–7.287; miR-499 rs3746444: TC+CC vs. TT: OR=0.690, 95%CI= 0.211–2.261, respectively). The results of meta-regression demonstrated that no overall significant heterogeneity was found in ethnicity, case-control match, and whether genotype distribution of controls was consistent with HWE or the sample size larger than 500 (**Table 2**). The results of Begg’s and Egger’ s tests were shown in **Table 3**, with the symmetrical distribution of effect sizes inside the Begg’s funnel plots (**Figure 5**), suggesting no significant publication bias among the included studies. Meta-regression and publication bias on miR-149 rs2292832 was not performed on account of insufficient studies.

**Table 2:** Summary of meta-regression analyses for heterogeneity ascertainment.

Covariate	miR-146a rs2910164				miR-196a2 rs11614913				miR-499 rs3746444			
	Coefficient	Std.Err	*t*	*P*	Coefficient	Std.Err	*t*	*P*	Coefficient	Std.Err	*t*	*P*
Ethnicity	-0.361	0.303	-0.12	0.907	-0.654	0.430	-1.52	0.159	-0.370	0.420	-0.73	0.480
HWE	-0.085	0.182	-0.47	0.650	-0.477	0.210	-2.27	0.047	0.275	0.286	0.96	0.355
Match	0.211	0.191	1.10	0.291	-0.196	0.259	-0.76	0.467	0.051	0.311	0.16	0.873
Sample size	0.119	0.204	0.58	0.572	-0.002	0.271	-0.01	0.992	0.240	0.296	0.81	0.434

Std.Err, Standard error.

**Table 3:** Summary of Begg’ s and Egger’ s tests.

SNP	Begg’s test		Egger’s test	
	Z	*P*	Z	*P*
miR-146a (rs2910164)	0.08	0.934	0.01	0.994
miR-149 (rs2292832)	-0.19	0.851	-1.79	0.655
miR-196a2 (rs11614913)	-0.15	0.882	0.95	0.352
miR-499 (rs3746444)	0.16	0.869	1.39	0.493

### Network Meta-Analysis

**Figure 6:**
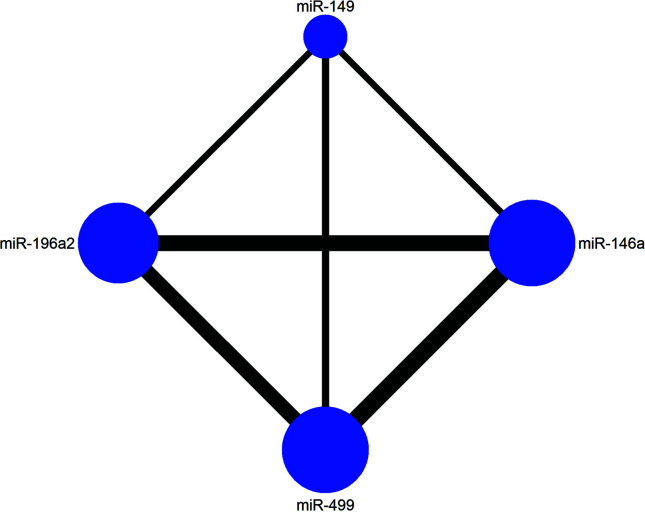
The network evidence plot of four single nucleotide polymorphisms (SNPs).

The current study contained four SNPs: miR-146a rs2910164, miR-149 rs2292832, miR-196a2 rs11614913, miR-499 rs3746444. It was observed from the network evidence that the number of direct comparisons of miR-146a vs. miR-499 was the largest, followed by miR-146a vs. miR-196a2, miR-196a2 vs. miR-499 (**Figure 6**). The predictive value of pairwise and network results of four miRNAs were explored and compared in **Table 4**. The NMA results showed no significant difference in the distribution of genotype frequencies except for miR-196a2 rs11614913, which appeared to have the highest superiority index when comparing and ranking four SNPs, further suggesting that it could be an effective indicator of the occurrence of HCC.

**Table 4:** The comparisons of pairwise and network results.

SNP	Comparisons	Pairwise meta-analysis		Network meta-analysis		S
		Direct OR	95% CI	Network OR	95% CI	
Case vs. control group						
miR-146a (rs2910164)	GC+CC vs.GG	1.003	0.904–1.113	1.02	0.73–1.38	0.89
miR-149 (rs2292832)	TC+CC vs.TT	0.898	0.756–1.068	0.96	0.47–1.79	0.96
miR-196a2 (rs11614913)	TC+CC vs.TT	1.232	1.028–1.476	1.20	1.01–1.43	2.31
miR-499 (rs3746444)	TC+CC vs.TT	1.197	0.973–1.472	1.20	0.96–1.47	1.00
HBV-positive vs. HBV-negative in case group						
miR-146a (rs2910164)	GC+CC vs.GG	1.687	0.667–4.263	1.62	0.77–2.87	1.40
miR-149 (rs2292832)	TC+CC vs.TT	2.435	0.116–51.063	2.45	0.51–7.53	2.65
miR-196a2 (rs11614913)	TC+CC vs.TT	3.005	1.239–7.287	2.46	1.31–4.09	2.34
miR-499 (rs3746444)	TC+CC vs.TT	0.690	0.211–2.261	0.96	0.38–2.01	0.54

OR, odd ratios; 95%CI, 95% confidence intervals.

## Discussion

MiRNAs play an important role in gene regulation of diseases (53), and have been proved to be tumor-suppressor genes as wells as oncogenes (54, 55). The dysregualtion of miRNA and its associated gene expression are involved in the occurrence and prognosis of HCC (56). The discovery of polymorphisms in miRNA genes has potential as new biomarkers for early diagnosis and prognosis in high-risk population, opening up new prospects for individualized treatment of HCC (57).

Of the 20 studies included in our research, the vast majority came from Asia, only two from Africa. Despite the rising incidence in North America and Europe, no enrolled studies came from either continent. The incidence of HCC varies widely within geographic locations. It is more common in low- and middle-income countries than in developed countries (58). It’s worth noting that, HCC incidence rates have been increasing in the United States, Europe and other developed areas (59). Obesity, smoking, diabetes, alcoholic cirrhosis and non-alcoholic steatosis are main causes of the increasing incidence of HCC (60–62). Currently, studies on the relationship between polymorphisms of miRNA and HCC are still lacking in relatively low-incidence areas. Our study illustrated the need for multi-ethnic, large-sample case-control studies that include data from a broad range of ethnic groups to obtain more stable and reliable results.

The pairwise results indicated that among the polymorphisms of miR-146a rs2910164, miR-149 rs2292832, miR-196a2 rs11614913, miR-499 rs3746444, only miR-196a2 was significantly associated with the susceptibility of HCC. When compared with TT genotype, CT or TT genotype in miR-196a2 carried a 1.232-fold increased risk of HCC. The network results were consistent with the direct results, with slight difference which was acceptable, indicating that our network evidence were robust. When comparing and ranking four SNPs, miR-196a2 rs11614913 appeared to have the highest superiority index. All of the above might come to the conclusion that miR-196a2 could serve as the best predictor of susceptibility in HCC.

HBV infection has been well established as one of the leading causes for the carcinogenesis of HCC (63). When comparing HBV-positive with HBV-negative HCC patients, a significant 3-fold increase in the frequencies of TC+CC versus TT was observed in miR-196a2 rs11614913. This indicated that the miR-196a2 rs11614913 polymorphism could be associated with the risk of HBV-related HCC. There have been studies that miRNAs could be involved in the development of HBV-related HCC. Previous reports have indicated that compared to normal liver, miRNA expression profiles were altered in chronic hepatitis B tissues (64). Wang et al. speculated that cellular miRNAs might function in HBV-related HCC, which affected HBV gene expression by binding to HBV transcripts or targeted cellular transcriptions factors that were necessary for HCC development (65). HBV infection could affect miRNA expression and contribute to enhanced viral replication and pathogenesis, and could ultimately lead to HCC (66).

MiR-196a2 rs11614913 is reported to be an important SNP associated with the etiology, progression and prognosis of several kinds of cancer. MiR-196a2 is located in the 3’passenger strand mature sequence of miR-196a2 (67), whose C to T mutation results in a G:C mutation to a G:U mismatch, leading to a decrease in the processing efficiency of the precursor of miRNAs to its mature form and ability to regulate target genes (68). The impacted expression level of the mature miR-196a2 can lead to genetic susceptibility and affect the survival of certain types of tumor. A number of studies has supported the proposition that the polymorphism of miR-196a2 rs11614913 may contribute to the susceptibility of several cancers (69–72). In the updated meta-analysis of Liu et al. (69), the link between miR-196a2 rs11614913 and a variety of cancers was explored and found that it was associated with HCC and lung cancer susceptibility. Hoffman’s research suggested that miR-196a2 had potential carcinogenic effects during the development of breast cancer (70). Hu et al. provided evidence that miR-196a2 variant homozygote was associated with a 1.76-fold-elevated HR, which was unfavorable to the overall survival of non-small cell lung cancer (71). In a case-control study conducted by Dikaiakos et al. (72), no significant association was found between miRNA-196a2 and colorectal cancer. Research has shown that the C allele of miRNA-196a2 increased the expression of mature miRNA-196a2 in HCC tissues (73). It is biologically plausible that miR-196a2 rs11614913 polymorphism may contribute to genetic susceptibility of HCC.

Although our results indicated that only miR-196a2 was associated with the susceptibility of HCC, and there was not enough evidence to support the association in miR-146, miR-149 or miR-499, the negative results still could not be ignored and should be interpreted cautiously. This is due to the occurrence of HCC is the result of multiple factors, in addition to complex genetic factors, there are hepatitis, aflatoxin exposure, Hepatitis C virus infection and other factors (74–76). Instead the incidence of HCC, gene variation may only cause increased susceptibility in a certain extent (77). Geography and ethnicity also need to be taken into account. Differences in populations are an important consideration in genetic association studies which may lead to inconsistent outcomes and difficulties in repetition (78, 79).

There are some limitations in our study. Only published studies were included, and those studies with negative results that could not be published were likely to be omitted, leading to incomplete studies. Secondly, selection bias could exist and impact on the results since the control group in most studies are hospital-based rather than population-based. Finally, although we found that miR-196a2 could be a potential indicator, how this might predispose to HCC are unclear and further functional studies are needed to clarify the mechanisms.

## Conclusion

In conclusion, we found that the genetic polymorphism of miR-196a2 rs11614913 is significantly associated with the occurrence of HCC, especially for HBV- related HCC, and that individuals with TC/CC allele were more susceptible. No significant association was found in miR-146a rs2910164, miR-149 rs2292832, or miR-499 rs3746444. Our work could provide important information on the relationship between these four miRNAs and the susceptibility of HCC, suggesting potential novel diagnostic options. This would contribute to the reduction of mortality through early screening and diagnosis and improve the efficacy in HCC management.

## Data Availability Statement

The original contributions presented in the study are included in the article/supplementary materials. Further inquiries can be directed to the corresponding author.

## Author Contributions

QZ conducted the design of study, extracted the data, performed statistical analysis, and wrote the initial manuscript after consultation with the other authors. XX and HL improved the design, revised the manuscript, and approved the final version. TQ and SW checked the preliminary data. MW participated in the quality assessment adjudication. All authors contributed to the article and approved the submitted version.

## Funding

This study was partly supported by the Social Sciences Foundation of Liaoning Province (No. L18ATJ001).

## Conflict of Interest

The authors declare that the research was conducted in the absence of any commercial or financial relationships that could be construed as a potential conflict of interest.
